# Importance of B Lymphocytes and the IgG-Binding Protein Sbi in *Staphylococcus aureus* Skin Infection

**DOI:** 10.3390/pathogens5010012

**Published:** 2016-01-27

**Authors:** Fan Zhao, Anita S. Chong, Christopher P. Montgomery

**Affiliations:** 1 Department of Pediatrics, The University of Chicago, Knapp Center for Biomedical Discovery 5124, 900 E. 57th Street, Chicago, IL 60637, USA; fzhao@peds.bsd.uchicago.edu; 2 Department of Surgery, The University of Chicago, Chicago, IL 60637; USA; achong@surgery.bsd.uchicago.edu

**Keywords:** MRSA, skin infection, Sbi, protein A, *Staphylococcus aureus*, antibody, protective immunity

## Abstract

Recurrent *Staphylococcus aureus* infections are common, suggesting that immunity elicited by these infections is not protective. We previously reported that *S. aureus* skin infection (SSTI) elicited antibody-mediated immunity against secondary SSTI in BALB/c mice. In this study, we investigated the role of humoral immunity and the IgG-binding proteins Sbi and SpA in *S. aureus* SSTI. We found that B lymphocyte-deficient μMT mice were highly susceptible to infection, compared with congenic BALB/c mice. Importantly, transfer of immune serum protected μMT mice, demonstrating an appropriate response to protective antibody. We found that deletion of *sbi*, but not *spa*, impaired virulence, as assessed by skin lesion severity, and that Sbi-mediated virulence required B lymphocytes/antibody. Furthermore, neither Sbi nor SpA impaired the elicited antibody response or protection against secondary SSTI. Taken together, these findings highlight a B lymphocyte/antibody-dependent role of Sbi in the pathogenesis of *S. aureus* SSTI, and demonstrate that neither Sbi nor SpA interfered with elicited antibody-mediated immunity.

## 1. Introduction

*Staphylococcus aureus* causes a wide range of infectious syndromes, including asymptomatic colonization, relatively minor skin and soft tissue infections (SSTI), and more severe infections, such as complicated SSTI, pneumonia, bone and joint infections, and sepsis [1]. *S. aureus* infections have become epidemic in the United States, highlighted by the emergence of the community-associated methicillin-resistant genotype USA300 [1]. Recurrent infections are common, suggesting that *S. aureus* infections frequently fail to elicit immunity that protects against subsequent infections [2], and the adaptive immune mechanisms that protect against recurrent *S. aureus* infection remain elusive.

T lymphocyte mediated-immunity is clearly important in defense against *S. aureus* infections, because patients with Hyper IgE Syndrome, who have defects in pathways controlling Th17/IL-17A mediated immunity, have high rates of recurrent *S. aureus* pneumonia and SSTI [3]. In addition, patients with poorly controlled HIV infection and low CD4+ T cell counts are at high risk for recurrent *S. aureus* SSTI, although there are other factors besides T cell lymphopenia that could contribute to this observation [4,5]. In contrast, a role for humoral immune defects in predisposing to recurrent *S. aureus* infections remains less well defined. Increased frequencies of SSTI and *S. aureus* infections in patients with the inherited antibody deficiency X-linked agammaglobulinemia or with the common variable immunodeficiency have been reported [6,7], although whether this association is due specifically to the inability to produce protective antibodies remains unclear. Additionally, confounding the notion that antibodies play a critical role in the protection against SSTI, are the observations that anti-staphylococcal antibodies are almost universally detected in the healthy human population yet a portion still develop SSTIs [8,9,10].

The genome of *S. aureus* encodes for several proteins that bind IgG, suggesting that *S. aureus* has evolved mechanisms to inhibit and/or interfere with antibody-mediated immunity. For example, staphylococcal protein A (SpA) acts as a B cell superantigen by binding to the V_H_3 Fab portion of the B cell receptor and triggering apoptosis of B cells [11]. A consequence of this activity is the ability of SpA to inhibit antibody responses against other *S. aureus* antigens, thus preventing the development of protective antibody-mediated immunity [12,13]. Consistent with this hypothesis, intravenous infection with a SpA deletion mutant elicited more robust protective antibody responses to non-SpA antigens, compared with an isogenic wild-type isolate [14]. Pauli *et al.* recently reported another mechanism of SpA-mediated immune evasion, whereby the superantigenic activity of SpA leads to an antibody response that is largely focused on SpA and limits responses to other *S. aureus* virulence factors that confer protection [15]. These findings suggest that the mechanisms by which SpA prevent protective immune responses may be complex and multifactorial.

While SpA has been shown to be an important virulence factor in multiple mouse models of *S. aureus* pneumonia and bloodstream infection [16,17,18], the importance of another IgG binding protein, called second binder of IgG (Sbi) is less clear [19,20]. SpA binds to the Fcγ domain of IgG thereby preventing the ability of IgG to bind to host FcγRs [21]. In contrast, Sbi has two Ig-binding domains and two domains that bind to complement component C3. A consequence of Sbi binding to IgG and C3 is the futile consumption of C3, a novel strategy for immune evasion that may involve the recruitment of plasmin to degrade recruited complement components [22,23,24].

We recently reported a mouse model of recurrent *S. aureus* SSTI, in which primary infection protects BALB/c, but not C57BL/6, mice against secondary infection [25]. This protection was dependent on both antibody-mediated immunity and the Th17/IL-17A pathway, and was inhibited by the Th1/IFNγ pathway. Because of the importance of antibody-mediated immunity, we hypothesized that B lymphocytes play an important role in innate and adaptive defenses in this model. We also hypothesized that SpA and/or Sbi would be important in virulence in primary SSTI and would interfere with the development of protective immunity. We report herein that B lymphocyte deficient μMT mice have increased susceptibility to primary *S. aureus* SSTI, but retain the ability to respond to adoptively transferred protective antibody. We also observed a role for Sbi, but not SpA, in the virulence of primary *S. aureus* SSTI. The importance of Sbi in the virulence of primary SSTI was dependent on B lymphocytes and/or antibody, because there was no effect of Sbi on virulence in μMT mice. Surprisingly, neither Sbi nor SpA inhibited protective immunity or antibody responses. Taken together, these findings highlight a B cell/antibody dependent role of Sbi in virulence, and unexpectedly demonstrate that these IgG-binding proteins do not interfere with protective immunity in this model of recurrent *S. aureus* SSTI.

## 2. Results

### 2.1. Increased Susceptibility of B Cell Deficient Mice to S. aureus SSTI

**Figure 1:**
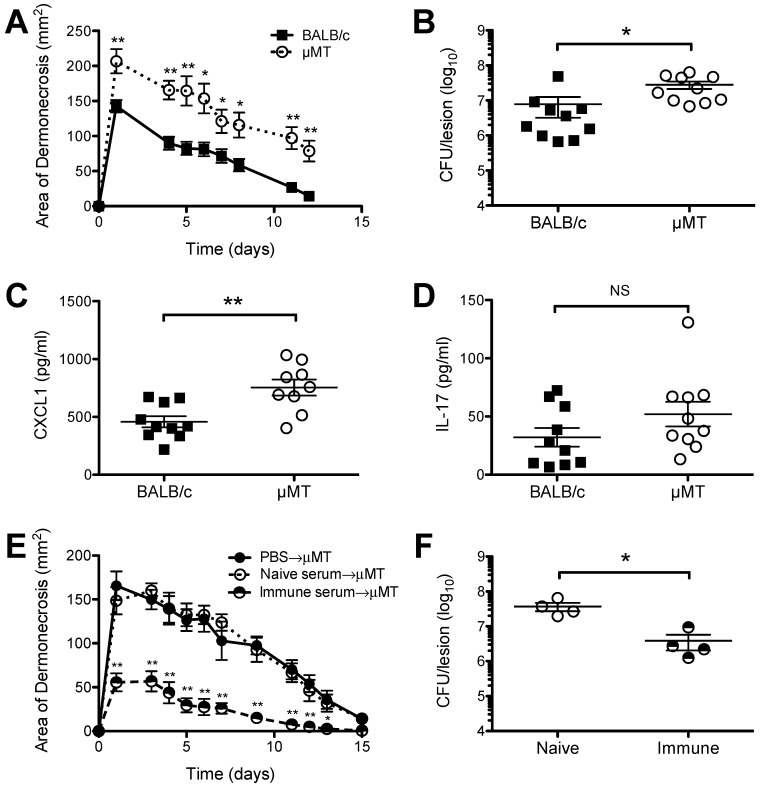
B cell deficient μMT mice had increased susceptibility to *S. aureus* SSTI, but responded to protective antibody. (**A**) μMT mice had larger lesions, compared with BALB/c mice, after primary *S. aureus* SSTI. The skin lesions of μMT mice had greater numbers of bacteria (**B**) and higher levels of CXCL1 (**C**) 3 d after infection, compared with BALB/c mice; (**D**) There were no significant differences in the levels of IL-17A in the skin lesions of BALB/c and μMT mice 3 d after infection; (**E**) Adoptive transfer of serum from previously infected BALB/c mice protected μMT mice, compared with transfer of serum from naive BALB/c mice or PBS; (**F**) There were fewer bacteria recovered from the lesions of μMT after transfer of immune BALB/c serum, compared with naïve serum. Results are presented as the means ±SEM (*n* = 4–10 mice/group). All experiments were performed at least twice. * indicates *p* < 0.05, ** indicates *p* < 0.01, NS indicates not significant.

To assess the role of B cells and/or antibodies in the defense against primary *S. aureus* SSTI (dermonecrosis), age-matched BALB/c and B cell deficient (μMT) BALB/c mice were inoculated subcutaneously with 1.5 × 10^7^ CFU *S. aureus* isolate 923. Subcutaneous inoculation of μMT mice resulted in significantly larger dermonecrotic lesions, compared with BALB/c mice (*p* < 0.01, Figure 1A). The larger lesions observed in μMT mice were associated with approximately 10-fold greater numbers of bacteria recovered from the lesions 3 d after infection (*p* < 0.05, Figure 1B), as well as higher levels of the inflammatory chemokine CXCL-1 (*p* < 0.01, Figure 1C), but not IL-17A (*p* = 0.15, Figure 1D). To determine whether μMT mice retained the ability to respond to protective antibody, serum from naïve or previously infected BALB/c mice was adoptively transferred to naïve μMT mice prior to SSTI. Transfer of immune BALB/c serum resulted in significantly smaller skin lesions, compared with recipients of PBS or serum from naïve mice (*p* < 0.01, Figure 1E). There were also fewer bacteria recovered from the lesions of μMT mice that received immune serum (*p* < 0.05, Figure 1F). The finding that there were fewer bacteria recovered from the lesions of WT mice, compared with μMT mice, together with our previous report of decreased numbers of bacteria recovered from the lesions of BALB/c mice following secondary SSTI, compared with primary SSTI [25], suggest that antibody-mediated immunity results in enhanced bacterial clearance from dermonecrotic skin lesions. Taken together, these findings suggested that B cells and the antibodies they produce play a role in defense against *S. aureus* SSTI by limiting bacterial growth and local production of the neutrophil chemoattractant CXCL-1, but μMT mice retained the ability to respond to adoptively transferred protective antibody.

### 2.2. A Role for Sbi, but not SpA, in the Pathogenesis of SSTI Caused by USA300

**Figure 2:**
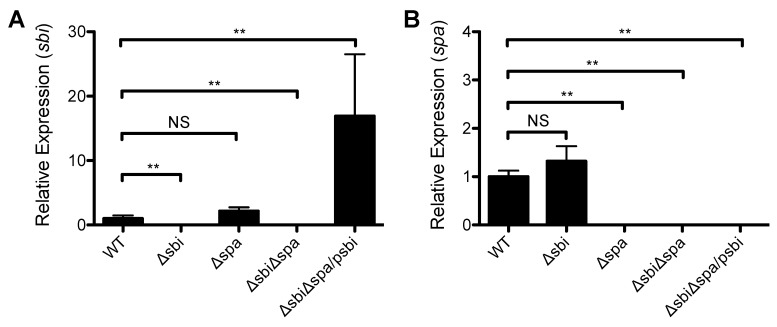
Expression of *sbi* and *spa* in the strains used in this study. (**A**) Expression of *sbi* by semi-quantitative RT-PCR in a wild-type *S. aureus* USA300 isolate (WT), isogenic *sbi* (*Δsbi*) and *spa* (*Δspa*) deletion mutants, a double *sbi*/*spa* deletion mutant (*ΔsbiΔspa*), and the double deletion mutant complemented with *sbi* expressed on a multi-copy plasmid (*ΔsbiΔspa*/p*sbi*); (**B**) Expression of *spa* by semi-quantitative RT-PCR. Expression is quantified by the ΔΔC_T_ method, normalized to a housekeeping gene (*gyrB*), and expressed relative to the WT strain. Results are presented as the means ± SEM. All experiments were performed at least twice. ** indicates *p* < 0.01, NS indicates not significant.

**Figure 3:**
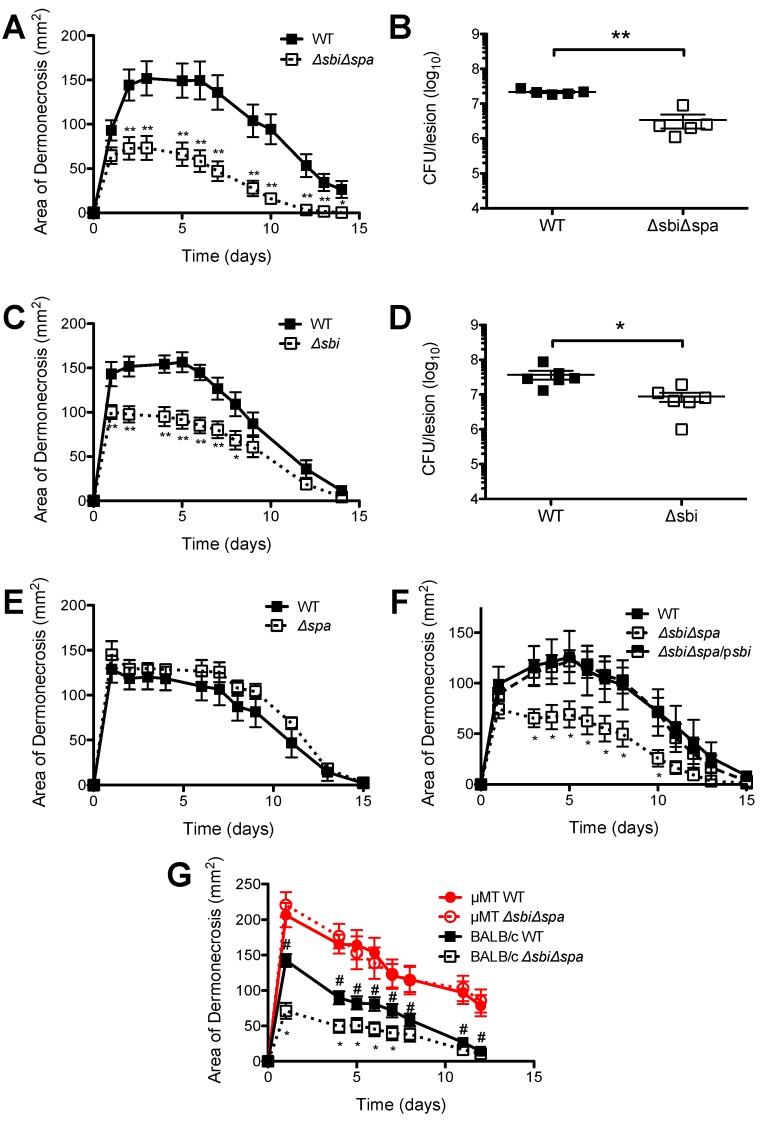
*Sbi*, but not *spa*, was a virulence factor in *S. aureus* SSTI and required B lymphocytes for virulence. Compared with infection with the wild-type USA300 isolate, infection of BALB/c mice with *ΔsbiΔspa* resulted in smaller skin lesions (**A**) and fewer bacteria recovered from the lesions 3 d after infection (**B**); (**C**,**D**) Infection with *Δsbi* also resulted in smaller skin lesions and fewer bacteria recovered from the lesions; (**E**) In contrast, there were no significant differences in lesion size afer infection with WT or *Δspa*; (**F**) Complementation of *sbi* into *ΔsbiΔspa* restored virulence; (**G**) There was no difference in the size of skin lesions in μMT mice infected with the WT USA300 isolate or *ΔsbiΔspa*. Results are presented as the means ± SEM (*n* = 5–10 mice/group). All experiments were performed at least twice. * indicates *p* < 0.05, ** indicates *p* < 0.01; For (**F**), * indicates *p* < 0.05 compared with BALB/c WT, # indicates *p* < 0.05 compared with μMT WT.

The increased susceptibility of μMT mice to *S. aureus* SSTI led us to hypothesize that the IgG binding proteins Sbi and/or SpA would promote the virulence of *S. aureus* SSTI. Therefore, the isogenic deletion mutants *Δsbi*, *Δspa*, or *ΔsbiΔspa* were constructed in strain 923. There were no significant differences in the growth rate of the strains (data not shown). Deletion was confirmed by semiquantitative RT-PCR (Figure 2). Subcutaneous inoculation of BALB/c mice with *ΔsbiΔspa* resulted in smaller lesions and a 0.8 log reduction in the number of bacteria recovered from the lesions, compared with inoculation with the wild-type isolate (WT) (*p* < 0.01, Figure 3A,B). To determine whether Sbi and/or SpA were necessary for virulence, mice were inoculated with the WT isolate, *Δspa*, or *Δsbi*. Consistent with our hypothesis, infection with *Δsbi* resulted in smaller dermonecrotic skin lesions (*p* < 0.01, Figure 3C) and fewer bacteria recovered from the lesions (*p* < 0.05, Figure 3D), compared with WT. In contrast, infection with *Δspa* resulted in no significant differences in the size of skin lesions compared with WT (*p* > 0.3, Figure 3E). Furthermore, complementation of *sbi* into *ΔsbiΔspa* restored virulence to that of the WT isolate (*p* < 0.05 compared with *ΔsbiΔspa*, Figure 3F). However, infection with *ΔsbiΔspa/psbi* did not restore the number of bacteria recovered from the lesions to levels observed with the WT isolate (1.6 × 10^7^ ± 4.0 × 10^6^ CFU after infection with *ΔsbiΔspa*; 1.0 × 10^7^ ± 3.9 × 10^6^ CFU after infection with *ΔsbiΔspa/psbi*; *p* = 0.3).

Because of the antibody-binding properties of Sbi, we hypothesized that Sbi mediated virulence via interaction with antibodies. To test this, we infected μMT mice with WT or *ΔsbiΔspa*. In support of this hypothesis, there were no significant differences in the size of dermonecrotic skin lesions between μMT mice infected with 923 or *ΔsbiΔspa* (*p* > 0.3, Figure 3G). Because the phenotypes of *Δsbi* and *ΔsbiΔspa* in this model are indistinguishable, we presume that the interaction with B cells and/or antibody is mediated by Sbi. Therefore, deletion of *sbi*, but not *spa*, attenuated virulence in this model of SSTI in BALB/c mice, and B lymphocytes and/or antibodies were required for Sbi-mediated virulence.

### 2.3. Neither Sbi nor SpA Impacted Protective Immunity against Recurrent S. aureus SSTI

**Figure 4:**
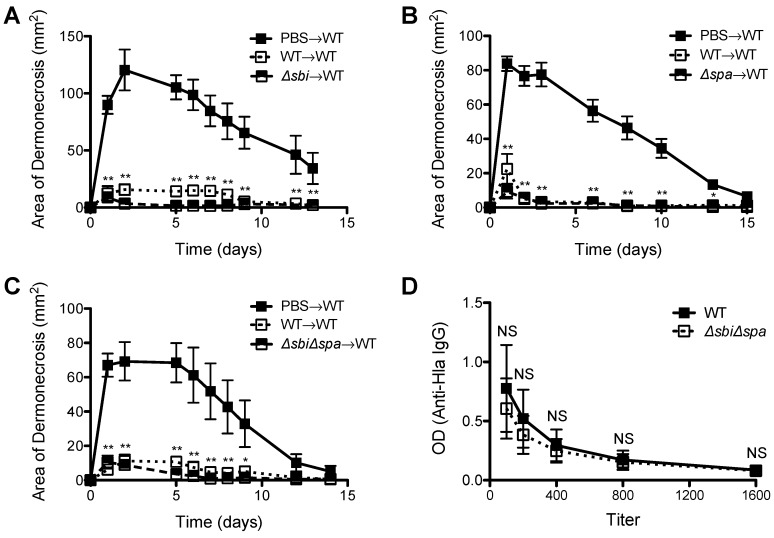
Expression of *sbi* or *spa* during primary *S. aureus* SSTI did not impact development of protective immunity in BALB/c mice. Primary SSTI with *Δsbi* (**A**); *Δspa* (**B**); or *ΔsbiΔspa* (**C**) protected BALB/c mice equally well against secondary SSTI with the WT isolate, compared with primary SSTI with WT; (**D**) There were no significant differences in anti-Hla IgG titers after infection with WT or *ΔsbiΔspa*. Results are presented as the means ± SEM (*n* = 5–10 mice/group). All experiments were performed at least twice. * indicates *p* < 0.05, ** indicates *p* < 0.01, NS indicates not significant.

Because of a demonstrated role for SpA in inhibiting the development of protective antibody responses against *S. aureus* sepsis [14], we tested the roles of Sbi and SpA in the development of protective immunity against *S. aureus* SSTI. We performed primary infection of BALB/c mice with WT, *Δsbi*, *Δspa*, or *ΔsbiΔspa*, and then 8 wks later challenged with WT *S. aureus*. Infection with WT, *Δsbi* (*p* < 0.01, Figure 4A), *Δspa* (*p* < 0.01, Figure 4B), or *ΔsbiΔspa* (*p* < 0.01, Figure 4C) all elicited protective immunity against secondary SSTI, suggesting that neither Sbi nor SpA affected the elicited antibody response in BALB/c mice. Furthermore, we quantified antibody levels against α-hemolysin (Hla), an important vaccine candidate [26,27] and observed no significant differences in anti-Hla IgG titers between mice infected with WT or *ΔsbiΔspa* (*p* > 0.4, Figure 4D).

**Figure 5:**
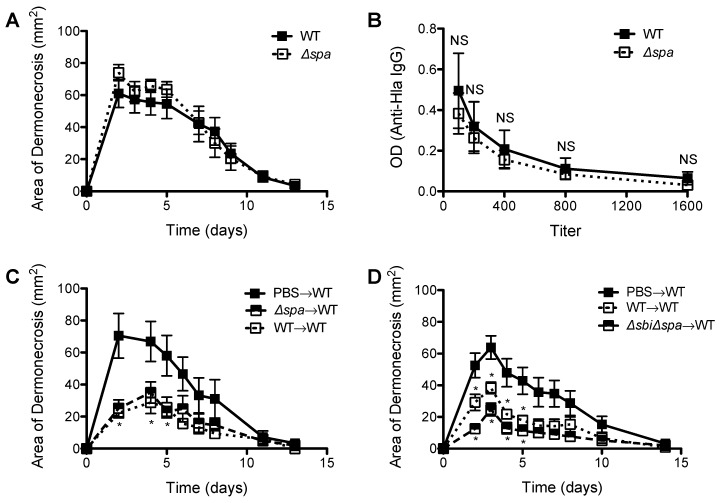
Expression of *spa* during primary SSTI did not impact protective immunity in C57BL/6 mice. (**A**) There were no signficant differences in the size of skin lesions after primary infection of C57BL/6 mice with WT or *Δspa*; (**B**) There were no significant differences in anti-Hla IgG titers after infection with WT or *Δspa*; (**C**) Primary SSTI with *Δspa* did not elicit superior protection, compared with the WT isolate, against secondary SSTI with WT in C57BL/6 mice; (**D**) Primary SSTI with *ΔsbiΔspa* did not elicit superior protection, compared with the WT isolate, against secondary SSTI with WT in C57BL/6 mice. Results are presented as the means ± SEM (*n* = 4–8 mice/group). All experiments were performed at least twice. * indicates *p* < 0.05 compared with PBS→WT, NS indicates not significant.

We previously reported that, in contrast to BALB/c mice, primary SSTI in C57BL/6 mice failed to elicit protective antibody-mediated immunity despite eliciting a polyclonal antibody response [25]. Because SpA has been reported to interfere with the repertoire of antigen-specific antibody responses [14], we hypothesized that primary SSTI with *Δspa* might be able to enhance protection in C57BL/6 mice. To test this, we performed primary SSTI in C57BL/6 mice with WT or *Δspa*, followed by assessment of anti-Hla IgG and secondary infection with WT. As we observed in BALB/c mice, there were no significant differences in the size of dermonecrotic skin lesions after primary infection with WT or *Δspa* (*p* > 0.3, Figure 5A). Furthermore, there were no significant differences in anti-Hla IgG titers of C57BL/6 mice after infection with WT or *Δspa* (*p* > 0.4, Figure 5B), suggesting no inhibition by SpA of potentially protective antibody responses. We also tested for the development of protective immunity. Although protection against WT SSTI in C57BL/6 mice after primary SSTI infection was considerably more modest than in BALB/c mice, we were nevertheless able to confirm that primary infection with *Δspa* elicited comparable protection against secondary SSTI with WT (*p* > 0.3, Figure 5C). There were also no significant differences in protection elicited after primary infection with WT or *ΔsbiΔspa* (*p* > 0.1, Figure 5D). Taken together, these findings suggest that neither Sbi nor SpA alter the magnitude of the antibody response to Hla and protective immunity against recurrent *S. aureus* SSTI.

## 3. Discussion

We found that B lymphocyte deficient μMT mice were highly susceptible to *S. aureus* SSTI, with larger dermonecrotic skin lesions, more bacteria in the lesions, and higher levels of the inflammatory chemokine CXCL-1 in the lesions, compared with congenic BALB/c mice. This is in contrast with reported findings in other models of *S. aureus* infection. For example, Spellberg *et al.* reported similar mortality after intravenous inoculation of *S. aureus* in BALB/c and μMT mice [28]. Similarly, Falugi *et al.* observed that bloodstream infection in μMT mice resulted in fewer bacteria in the kidneys and fewer bacterial abscesses, compared with wild-type mice [13]. *Rag2* deficient mice, that lack mature T and B cells, also did not have altered susceptibility in bloodstream model of *S. aureus* infection [29]. μMT mice also did not have impaired clearance of *S. aureus* in a model of nasal colonization [30]. Together, these and our current findings demonstrate that μMT mice are selectively susceptible to *S. aureus* SSTI, and suggest site-specific differences in the role of B cells in defense against *S. aureus*.

The differences in the resistance to primary infection in µMT and WT mice were observed by day 3 post-infection. The demonstration that larger skin lesions also had increased numbers of bacteria recovered suggests a defect in bacterial clearance. Although the kinetics of bacterial clearance and the role of bacterial burden in the severity of dermonecrotic lesions remains to be fully elucidated [31,32], we and others have demonstrated that large differences in lesion severity (e.g., WT *vs.* µMT mice and strain 923 *vs.* *Δsbi* or *ΔsbiΔspa* in this study) are accompanied by differences in bacterial burden and local inflammatory responses [25,33,34,35]. While the rapid kinetics suggested a role for nonspecific natural antibodies in defense against *S. aureus* SSTI, our findings that transfer of naïve BALB/c serum did not restore protection in μMT mice, whereas immune serum did, suggested instead for a role of elicited *S. aureus*-specific antibodies. These findings are consistent with an emerging role for B lymphocytes in early antibacterial defense, either through B cell production of Type I interferon and chemokines [36], and/or the early production of elicited antibodies. These findings have clinical implications for therapeutic passive antibody transfer into patients with B cell immune deficiencies.

SpA and Sbi are the two major IgG-binding proteins in *S. aureus*. By using *S. aureus* mutants lacking *spa* or *sbi*, we showed that Sbi, but not SpA, was important in the pathogenesis of primary *S. aureus* SSTI. The finding that SpA did not play a role in virulence is consistent with the recent findings that deletion of *spa* impacts skin abscesses, but not dermonecrosis, in a mouse model [37]. SpA also is not required for keratinocyte invasion [38]. These observations, together with reports of SpA being an important virulence factor in mouse models of *S. aureus* pneumonia [16,17] and sepsis [14], underscore the notion that the type of immune response that is protective depends on the site of *S. aureus* infection. In contrast to SpA, the role of Sbi in virulence is less well documented. A *spa/sbi* deletion mutant was reported to have attenuated virulence in an epicutaneous model of *S. aureus* infection [39]. In this study we demonstrate the importance of Sbi in virulence and its requirement of B cells/antibody, because differences between the virulence of WT and *ΔsbiΔspa* were lost in μMT mice. It is important to note that, although infection with the *sbi* deletion mutant resulted in fewer bacteria recovered from the skin lesions, complementation of the *sbi/spa* deletion mutant with *sbi* did not restore the number of bacteria in the lesions to a level comparable to infection with the WT isolate. These findings raise the possibility that Sbi acts, at least in part, by modulating local inflammatory responses, and not entirely by enhancing bacterial clearance. It is also possible that Sbi and SpA act synertistically to impact bacterial clearance. While we did not define how Sbi confers virulence, we speculate that because Sbi can bind to antibody and complement component C3, it can interfere with antibody/complement-mediated opsonphagocytosis [13]. More work will be needed to better understand the importance of surface-bound *vs.* secreted Sbi in the pathogenesis of *S. aureus* infection.

Our observations that *spa* and/or *sbi* expression did not affect the development of the antibody response against Hla contrast with recent reports that SpA interfered with elicited protective antibody responses both in mice and in humans [14,15]. In mice, immunization with a *S. aureus* isolate expressing a SpA mutant that could no longer bind to the Fcγ or Fab V_H_3 domains of IgG resulted in superior antibody levels against other *S. aureus* antigens and protection against *S. aureus* sepsis, compared with immunization with WT *S. aureus* [12,13,14]. In humans with *S. aureus* SSTI, Pauli *et al.* demonstrated that the elicited plasmablast/antibody response is skewed towards SpA [15]. This immunodominant effect of SpA prevented antibody responses against other protective antigens [15]. We reconcile these discrepant findings by hypothesizing that the quality of the elicited antibody response is highly dependent on the primary infectious syndrome. This hypothesis is supported by the fact that bloodstream infection with wild-type *S. aureus* does not elicit protective immunity [14], whereas SSTI does [25]. Therefore, we speculate that neither Sbi nor SpA inhibits antibody responses against Hla or other *saeRS*-regulated antigens that mediate protection against secondary SSTI [40], but probably do so in other models. The mechanistic details on how SpA inhibits antibody responses in some but not other models of *S. aureus* infection requires further investigation.

In summary, we found that B cell deficient μMT mice exhibited increased susceptibility to *S. aureus* SSTI that was not reversed by passive transfer of naïve antibody, but was restored by passively transferred immune sera. We also showed that the IgG binding protein Sbi, but not SpA, was important for virulence in *S. aureus* SSTI in primary infections, and that this virulence required the presence of B cells and/or antibody. Notably, neither Sbi nor SpA interfered with the elicited antibody response or protective immunity against secondary SSTI in BALB/c or C57BL/6 mice. Taken together, these findings highlight an important role for B cells, antibodies, and Sbi in innate defense against primary *S. aureus* SSTI, and the critical role of antibodies in protective immunity.

## 4. Materials and Methods

### 4.1. Bacterial Isolates

Strain 923 is a previously described USA300 clinical isolate from a patient with a skin infection at the University of Chicago [41]. *Δsbi* is an isogenic *sbi* deletion mutant of strain 923 generated by allelic recombination using the pMAD vector [42], in which the *sbi* gene has been replaced by the *aad9* gene, encoding spectinomycin resistance. *Δspa* and *ΔsbiΔspa* were generated by phage transduction of a gene encoding tetracycline resistance from 8325-4*Δspa*, a gift from Mathias Herrmann (University of Saarland) [43], into 923 and *Δsbi*, respectively (Table 1). Strain *ΔsbiΔspa/psbi* was constructed by transforming ΔsbiΔspa with a modified pCN48 in which expression of *sbi* was constitutively driven by the Pspac promoter (Table 1) [44].

**Table 1:** Oligonucleotides used in this study.

Gene	Sequence (5′-3′)	Application	Reference
*sbi* (downstream flanking region)	F—AAAGAATTCTCAATCAAAAATATCTTCTCT	deletion	This work
	R—AAACCATGGATATATAATAATCCATTT		
*sbi* (upstream flanking region)	F—AAAGGATCCATTAGTATAGTAACAATATTATG	deletion	This work
	R—AAAGAATTCGTGTATTCCCTTTCTTTT		
*aad9*	F—AAAGAATTCATCGAATCCCTTCGTGAGCG	deletion	[33]
	R—AAAGAATTCTAATAAACTATCGAAGGAAC		
*sbi*	F—AAAGGATCCATGAAAAATAAATATATC	complementation	This work
	R—AAAGAATTCTTATTTCCAGAATGATAA		
*sbi*	F—GGGCAGCAACAATTACGTTAG	qRT-PCR	This work
	R—TGTTTGGTGCTTAGTTGAAGTTTG		
	Probe—TGGGGAAGCAAAAGCGAGTGAAAAC		
*spa*	F—TTTGTCAGCAGTAGTGCCGTTTGC	qRT-PCR	[33]
	R—GGCAACAAGCCTGGCAAAGAAGAT		
	Probe—AAATGGGACGTCCAGCTGTCGAAGTT		

Note: restriction sites are underlined.

### 4.2. Expression Analysis by Semiquantitative Reverse-Transcription PCR (qRT-PCR)

To confirm the deletion of *sbi* and *spa* and complementation of *sbi*, frozen stocks of the bacterial isolates were subcultured onto tryptic soy agar (TSA) overnight at 37 °C. The following evening, one colony was subcultured into 5 mL tryptic soy broth (TSB) and grown overnight at 37 °C with shaking (250 rpm). The following morning, the overnight culture was diluted 1:100 in fresh TSB and grown to an OD_600_ of 0.7. The bacteria were pelleted by centrifugation and immediately frozen at −80 °C. For RNA isolation and purification, the pellets were thawed on ice, resuspended in Tris-EDTA, and lysed with the addition of lysostaphin (200 μg/mL) at room temperature for 10 min. Buffer RLT was added and RNA purification was performed using the RNeasy kit with on column DNase treatment (Qiagen). For each sample, 2 μg RNA was reverse transcribed using the High Capacity Archive cDNA kit (Applied Biosystems). qRT-PCR was performed using Prime Time™ qPCR primer probe mixes (Integrated DNA Technologies) for *sbi* and *spa*, with *gyrB* as an endogenous control (Table 1). Relative quantification was calculated by the ΔΔC_T_ method, with expression of each gene in strain 923 as the reference.

### 4.3. Mouse Models of Primary and Secondary S. aureus SSTI

All animal experiments were conducted under protocols approved by the Institutional Animal Care and Use Committee at the University of Chicago. The mouse models of primary and secondary *S. aureus* SSTI have been described [25,33]. Female BALB/c and C57BL/6 mice were purchased from Taconic and were infected at 7 weeks of age. Female μMT BALB/c mice (Igh-Jtm1Dhu) were purchased from Taconic or bred at the University of Chicago. Briefly, on the day of inoculation, an overnight culture of *S. aureus* was diluted 1:100 in fresh TSB and grown to an OD_600_ of 1.8. The bacteria were centrifuged and washed twice in sterile phosphate buffered saline (PBS), after which they were resuspended in PBS to achieve a concentration of 1.5 × 10^7^ CFU/50 μL. Prior to inoculation, all mice were sedated by administration of ketamine and xylazine. For the model of primary SSTI, the right flank was shaved with clippers and 50 μL of *S. aureus* (1.5 × 10^7^ CFU) was injected subcutaneously. For the model of secondary SSTI, inoculation on the left flank was performed 8 weeks after primary infection. The lesion severity was assessed by digitally measuring the area of dermonecrosis (Adobe Photoshop) for 15 d after infection.

### 4.4. Quantification of Bacteria and Cytokines in the Skin Lesions

Groups of mice were sacrificed 3 d after infection by CO_2_ inhalation. Following sacrifice, the skin lesions were removed aseptically and placed into sterile PBS. The lesions were homogenized and an aliquot of homogenate was removed, serially diluted, and plated on mannitol salt agar for CFU quantification. Colonies were counted 24 h after plating. For measurement of cytokines, the homogenate was centrifuged and the supernatant was immediately frozen at −80 °C. CXCL-1 and IL-17 were quantified in the supernatants using commercially available ELISA kits (R & D Systems, Minneapolis, MN, USA).

### 4.5. Serum Transfer

BALB/c mice were sacrificed 14 d after secondary SSTI with *S. aureus* strain 923 (or PBS) by CO_2_ inhalation. Blood was obtained by cardiac puncture and serum was isolated using serum separator tubes (BD Biosciences, Franklin Lakes, NJ, USA). Adoptive transfer of serum was performed by retroorbital injection (100 μL) on each of the 2 d prior to infection.

### 4.6. Assessment of Antibody Levels by ELISA

EIA/RIA 96-well plates (Costar, Corning Inc., Corning, NY, USA) were coated with 5 μg/mL α-toxin (Hla) (Sigma-Aldrich, St. Louis, MO, USA). Mouse serum was prepared from whole blood using serum separator tubes (BD Biosciences). The serum was serially diluted in PBS and added to the antigen containing wells. Detection was performed using alkaline phosphatase (AP)-conjugated goat anti-mouse IgG, (1:5000; AffiniPure, Jackson ImmunoResearch, West Grove, PA, USA) and AP substrate p-NitroPhenyl Phosphate (pNPP) (Sigma-Aldrich). Absorbance was measured using a GENios spectrophotometer (Tecan, Männedorf, Switzerland).

### 4.7. Data Analysis

The size of the skin lesions were compared using student’s t test or one-way ANOVA with Tukey’s post test, where appropriate. The bacterial CFU and cytokine levels in the skin lesions and anti-Hla levels were compared using student’s t test. The expression of *sbi* and *spa* by qRT-PCR were compared using one-way ANOVA with Tukey’s post test. All statistical analyses were performed using GraphPad Prism. *p* < 0.05 was considered statistically significant.
